# Surgical management of chronic proximal hamstring tendinopathy in athletes: a 2 to 11 years of follow-up

**DOI:** 10.1007/s10195-013-0226-2

**Published:** 2013-02-09

**Authors:** Francesco Benazzo, Matteo Marullo, Giacomo Zanon, Cristian Indino, Francesco Pelillo

**Affiliations:** 1 Clinica Ortopedica e Traumatologica, IRCCS Policlinico San Matteo, Viale Golgi 70, 27100 Pavia, Italy; 2 Università degli Studi di Pavia, Pavia, Italy; 3 Dipartimento di Scienze Biomediche per la Salute, Università degli Studi di Milano, Milan, Italy

**Keywords:** Hamstring injuries, Proximal hamstring tendinopathy, Sport injuries, Lower limb surgery, Muscle injuries

## Abstract

**Background:**

Proximal hamstring tendinopathy typically afflicts athletes. The poor knowledge of this pathology can lead to late diagnosis and late treatment, which in chronic cases could be challenging. Surgical treatment could resolve the symptoms and could permit the return to full sport activity also in chronic cases.

**Materials and methods:**

We retrospectively evaluated 17 high-level athletes surgically treated for proximal hamstring tendinopathy. Symptoms lasted for an average of 23 months and were resistive to conservative treatment.

**Results:**

The follow-up period averaged 71 months. Return to run without pain occurred at a mean of 2.4 months (range 1–4) after surgery. All patients returned to sports at their pre-symptom level at a mean of 4.4 months after surgery. Results were excellent in 15 patients (88 %) and good in two patients (12 %). No results were fair or poor.

**Conclusions:**

Surgical treatment to manage chronic proximal hamstring tendinopathy in high-level athletes showed excellent results in terms of relief from symptoms and return to previous sport level.

## Introduction

Proximal hamstring tendinopathy (PHT) was firstly described by Puranen and Orava in 1988 with the name of “hamstring syndrome” [1]. The mean feature of PHT is sub-gluteal pain occurring during sport activity, stretching or in a prolonged sitting position, which can radiate down to the posterior thigh till the popliteal fossa. Hamstring weakness is often associated. Running abilities, particularly sprinting and acceleration, are impaired. The pain typically appears and gradually increases without reports of any trigger events [2].

On clinical examination, there is sometimes a deep focal tenderness at the ischial tuberosity. Active stretch tests of the posterior thigh recreate a sensation of tightness or pain at the ischial tuberosity. Peripheral neurological tests are typically normal, no weakness is reported in knee flexion and hip extension [3, 4].

Hamstring muscles overuse or previous acute hamstring strains are a common background. In most serious cases the consequent fibrosis can tether the sciatic nerve to the hamstring muscles leading to its irritation, compression or traction [2].

The typical magnetic resonance imaging (MRI) findings of PHT are increased signal intensity on T1-weighted and proton-density images of proximal hamstring tendon intrasubstance, an increased tendon girth on axial views and asymmetric involvement of hamstring tendon in unilateral cases [2, 5].

Biopsy specimens from the hamstring tendon of patients with PHT showed typical morphologic findings of tendinosis (rounding of tenocyte nuclei, increased ground substance, collagen disintegration, and increased vascular proliferation), absence of fibrocartilagineous metaplasia or calcification and no inflammatory cells [3].

The PHT is often misdiagnosed; also even when diagnosis is made, decision-making can be challenging.

This condition could frustrate the patient, who is often a high-level sportsman and needs to return to competition as fast as possible.

As with other tendinopaties, the first option of treatment is nonoperative and includes: modified or complete rest from sporting activity, stretching of the hamstrings, local and systemic nonsteroidal anti-inflammatory drugs (NSAIDs), physiotherapy, shockwave therapy, eccentric strengthening, and local steroidal injections [6–8]. Surgery is performed when conservative treatment fails.

The goal of this study is to present the long-term results of delayed surgical treatment for chronic PHT in elite athletes, hoping to help clinicians to manage the decision-making process.

## Materials and methods

We performed a retrospective chart review of 17 sportsmen presenting for surgical treatment of chronic PHT by the senior author between January 2000 and July 2009. Twelve patients (71 %) were men and 5 (29 %) were women. The average age was 26.6 years (range 20–34, median 26, SD 3.58). Nine patients (53 %) were professional athletes at the international level, with an average age of 25.0 years (range 20–30, median 25, SD 2.53); eight (47 %) practiced sport at a competitive level, with an average age of 28.5 years (range 23–34, median 29, SD 3.67). Thirteen (76 %) were in track and field sports, two (12 %) were long-distance runners and two (12 %) were footballers (Table 1). The right side was affected in six patients (35 %), the left side in 11 (65 %); there was only one bilateral case, but the second side was not considered in this study because of the follow-up inferior to 24 months.

**Table 1:** Sport activities and level of competition of the 17 athletes

Activity	Professional athletes	Competitive level athletes
Hurdles	3 (18 %)	3 (18 %)
Middle distance running	2 (12 %)	2 (12 %)
Triple jump	2 (12 %)	1 (6 %)
Soccer	2 (12 %)	
Long-distance running		2 (12 %)
Total	9 (53 %)	8 (47 %)

Before surgery, a clinical examination and a Magnetic Resonance Imaging (MRI) of the thigh were routinely performed; we additionally asked the patients about their medical history and previous diagnoses. After surgery, a questionnaire, submitted through interviews at the time of follow-up, allowed us to collect further information about: time needed for walking, for running without pain and for return to competitive activity, recurrences and patient’s satisfaction. Pain was assessed by VAS (Visual Analogic Scale): from 0 (no pain) to 10 (maximum pain experienced). Results were classified as excellent, good, fair and poor according to residual symptoms and return to sports. The result was considered excellent if the patient was asymptomatic and returned to the same pre-symptom level of sports; good if he felt pain during intense efforts, but anyway he returned to his previous level of participation. The result was fair if symptoms persisted after surgery and the athlete was forced to a lower level of participation, and poor if the patient was unable to return to sport.

The study conforms to the Declaration of Helsinki and was approved by the institutional committee for medical ethics. All patients provided informed written consent.

### History and clinical findings

All patients had chronic pain in the lower gluteal region, mostly at the site of the ischial tuberosity; four patients (23 %) referred pain radiating along the posterior aspect of the thigh down to the popliteal fossa. Sport evoked pain in 14 patients (82 %): nine by sprinting and five also by running at constant speed; three patients (18 %) had pain irrespective of sport activity. Eight patients (47 %) felt pain also during a prolonged sitting position. At the time of clinical examination, symptoms involved only one side in all cases. Clinical assessment is resumed in Table 2.

**Table 2:** Preoperative clinical evaluation

	No tenderness	Ischial tuberosity	Distal to ischial tuberosity
Tenderness	6 (35 %)	7 (41 %)	5 (29 %)

	No symptoms	Pain	Weakness
Concentric Hamstring Contraction	5 (29 %)	8 (47 %)	8 (47 %)

	Non Limited	Limited
Range of motion of Hip and Knee	14 (82 %)	3 (18 %)

	Positive	Negative
Leg Raising Test	4 (23 %)	13 (76 %)
Puranen-Orava test	15 (88 %)	2 (12 %)
Neurologic tests	0 (0 %)	17 (100 %)

There are no internationally validated score for PHT. We consider the return to preoperative level of activity as the best evaluation of the treatment proposed. The average pre-symptoms Tegner Score was 7.8 (range 7–10, SD 0.8); the average preoperative Tegner Score was 4.8 (range 4–6, SD 0.5).

The average VAS score for pain was 6.9 (range 4–8, SD 1.2). Pain appeared and increased gradually without any acute event; however, 13 patients (76 %) had almost one previous acute hamstrings strain occurringat an average of 7.9 months (range 6–10) before the onset of symptoms. These lesions were documented by ultrasonography at the time of injury; they involved the bellies of the biceps femoris muscle in seven cases, the belly of the semitendinosus muscle in two cases, and the semimembranosus in four cases.

### Instrumental examinations

**Fig. 1:**
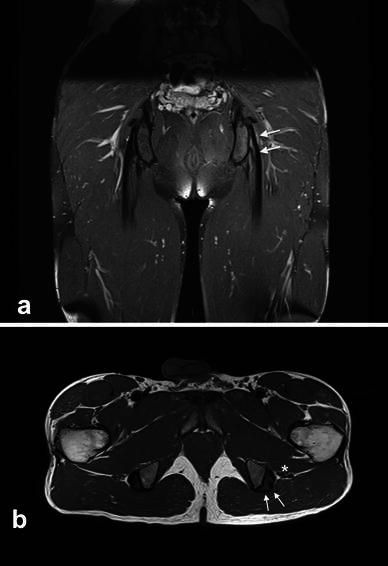
Magnetic resonance images of a 27 years old male hurdler with chronic posterior left sub-gluteal pain. **a** Proton density-weighted coronal image showing tendinosis of proximal left semimembranosus tendon (*arrows*). **b** T1-weighted axial image clear defines tendinosis and not tear (*arrow*). Left sciatic nerve runs immediately lateral to the tendon (*asterisk*)

In all patients, the pre-operative MRI of the thigh showed: signal alterations of hamstring tendons or bellies compatible with scars of distractive injuries, thickening or edema of the tendons, irregularity in the morphology of the muscle fibers and of the perineural tissue of the sciatic nerve (Fig. 1). The common hamstring tendon was involved in four patients (23 %), in one of the biceps femoris muscle in seven cases (41 %), in the semimembranosus in five cases (29 %) and in the semitendinosus in one patient (6 %).

Six patients (35 %) had also ultrasonography; in all cases could be recognized scars of previous distractive injuries of the hamstring, but it didn’t clearly define them. Electroneuromyography (ENMG) was done in 14 patients (82 %), showing no abnormalities in sciatic nerve conduction.

### Conservative treatment

All patients were initially treated conservatively elsewhere with various non-surgical methods.

Conservative therapy consisted of:

- local steroidal injections in seven patients (41 %);
- physical therapy (Tecar, laser, ultrasound) in eight patients (47 %);
- other physiotherapeutic techniques (soft-tissue mobilization, isometric exercises of the gluteus, quadriceps femoris and hamstrings) in 12 patients (71 %);
- local and systemic NSAIDs in five patients (29 %);
- eccentric contraction exercises of hamstrings in all cases.

The average duration of conservative treatment was 4.5 months (range 3–6, SD 1.05).

### Surgical treatment

Surgical treatment was performed if:

- Symptoms caused limitation or interruption of sport participation;
- MRI findings were suggestive for hamstring tendinopathy or scars of their previous tears;
- Conservative treatment was not effective.

**Fig. 2:**
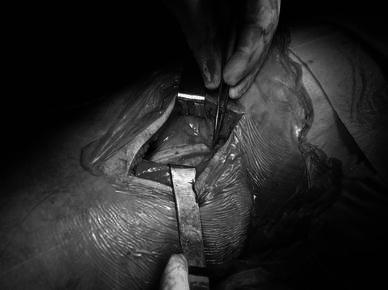
Intraoperative photograph. After section of the fascia superficialis, the distal edge of the gluteus maximus muscle is retracted; the proximal hamstring tendons are exposed

**Fig. 3:**
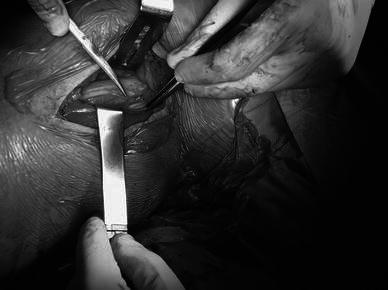
Intraoperative photograph. The sciatic nerve is identified laterally to the hamstring tendons and freed from adhesion

The average time between the onset of symptoms to surgery was 23 months (range 3–48, SD 10.0). This period lasted less than one year in three patients (18 %), between 1 and 2 years in eight patients (47 %), between two and three years in four patients (23 %) and between three and four years in two patients (12 %). Surgery was performed with the patient prone and the leg flexed to relax the hamstring muscles and the sciatic nerve; incision started from the ischial tuberosity extending 8–15 cm distally. The posterior cutaneous femoral nerve was carefully spared and the superficial fascia sectioned. The distal edge of the gluteus maximus muscle was reached and retracted superiorly; the proximal insertion of hamstring muscles was then exposed (Fig. 2). The tendon involved in the scarring process appeared hypertrophic and markedly fibrotic; we performed its partial section or multiple punctures in order to relax the myotendinous unit. The tendon involved was the one of biceps femoris muscle in nine patients (53 %), the semimembranosus in five patients (29 %) and the semitendinosus in one patient (6 %). In two patients (12 %) the tendon involved in the fibrotic reaction was not precisely detectable. In one patient adhesions also involved the ischium; they were freed and the ischium was drilled in order to revitalize the suffering enthesis. No hamstring ruptures were found. Fibrous tissue was present between the hamstring muscles medially and the sciatic nerve laterally. Sciatic nerve release was routinely performed, starting from the point where hamstrings cross the gluteus maximus muscles and continuing 10 cm down maximum (Fig. 3).

### Post-operative management

Continue passive motion of the hip and knee joints and stretching of the hamstrings starting immediately. Active motion was encouraged from the first postoperative day. Gradual recovery of full weight bearing was performed during the first ten days as tolerated. Antithrombotic prophylaxis with enoxaparin lasted for ten days. Progressive eccentric strengthening of hamstrings, gluteus and quadriceps, performed in open kinetic chain was set seven days from surgery. Swimming is allowed three weeks after surgery. After four weeks concentric strengthening in closed kinetic chain and bicycling started. Running was allowed two months after surgery.

## Results

**Fig. 4:**
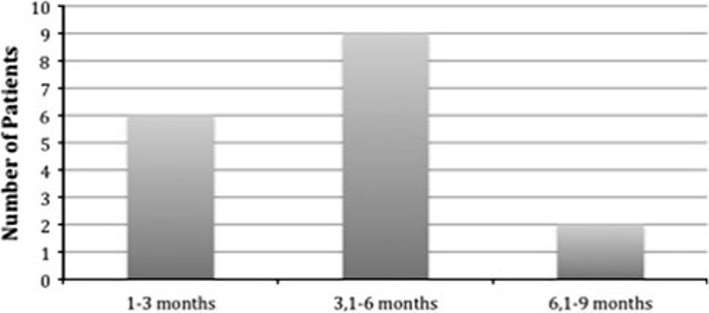
Time to return to sport activities after surgery

The average follow-up was 71.3 months (range 24–138 months, SD 30.7). Results were classified as excellent in 15 patients (88 %) and good in two patients (12 %). There were no fair or poor results. No recurrence was reported. Return to walk without pain occurred at an average time of 22 days after surgery (range 10–40, SD 6.6). Stretching exercises were free from pain at a mean of 36 days after surgery (range 21–60, SD 9.2). Running without pain occurred at a mean of 2.4 months after surgery (range 1–4, SD 1.0) (Table 3). Complete return to sport occurred at a mean of 4.4 months (range 2–9, SD 1.9) (Fig. 4). All patients returned to sports at their pre-symptom level; at the last follow-up the average Tegner score was 7.8 (range 7–10, SD 0.8), the same recorded before symptoms started.

**Table 3:** Postoperative time to return to activities

	0–15 days	16–30 days	31–45 days	46–60 days	61–90 days	91–120 days
Walking without pain	6 (35 %)	10 (59 %)	1 (6 %)			
Stretching without pain		4 (23 %)	11 (65 %)	2 (12 %)		
Running without pain		2 (12 %)	2 (12 %)	3 (18 %)	6 (35 %)	4 (23 %)

A questionnaire was submitted to rate patient’s satisfaction about the results of surgical treatment on a scale from 0 to 10, in which 10 indicated complete satisfaction. The average score was 9.1, ranging from 7 (one patient, 6 %) to 10 (eight patients, 47 %). Complications occurred in two patients (12 %). There was one (6 %) immediate complication (occurring in the first week after surgery), consisting in postoperative hematoma. It required surgical drainage 24 h after the first operation. The same patient had also a slower wound healing, treated with prolonged medications and it wasresolved in three weeks. The rehabilitation program was delayed about two weeks. A delayed complication occurred once (6 %); it consisted in hyperesthesia of the skin around the surgical scar, spontaneously clearing up in six weeks.

**Fig. 5:**
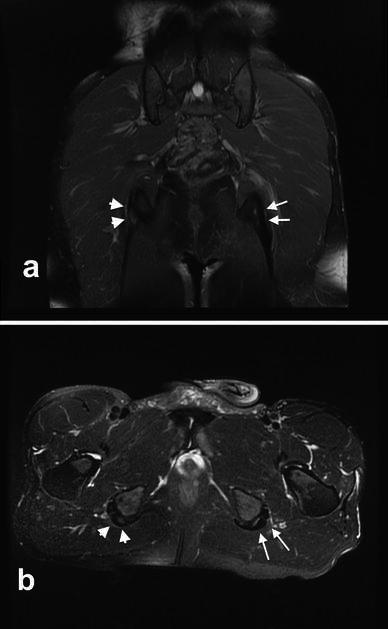
MRI of a 30 years old male long-distance runner 18 months after surgical treatment for left PHT. **a** Proton density-weighted coronal image showing no abnormal signal intensity of the left proximal hamstring insertion (*arrows*). The right proximal hamstring tendons present increased signal intensity compatible with PHT (*arrowheads*). **b** T2-weighted axial image clear shows no intratendinous structural abnormalities in the left proximal hamstring tendons (*arrows*). The right side presents signs of PHT (*arrowheads*). This patient underwent surgical treatment for right PHT four months after having performed the MRI, but he was not considered in this study because of the follow-up inferior to 24 months

The post-surgical MRI findings are shown in Fig. 5.

## Discussion

The most important finding of our study is that surgical treatment of PHT in competitive and high-level athletes permits return to sports at the same pre-symptom level as also in chronic situations. In our series it occurred on an average of 4.4 months, a good result comparable with recent literature [3, 9]. Complications after surgical treatment were common (12 %), but easily resolvable and they did not affect the return to sports.

Another important finding is that previous hamstring strains are a common background of PHT. In our series, 76 % of patients had previous hamstring tears; others studies reported lower percentages, ranging from 19 to 34 % [3, 4, 9]. Hamstrings are bi-articular, so they are particularly susceptible to injuries during high speed running, decelerating activities involving eccentric contractions or countermovement (hell kicking) and stretching at extreme joint positions (maximum hip flexion combined with knee extension) [4, 5]. Hamstrings strains are common injuries in various sports, particularly running, track and field and football [10, 11]. Ekstrand reported hamstring strains accounting for 12 % of all football injuries, concluding that their prevalence is increasing [10]. So, PHT could become more and more relevant in orthopaedic sports traumatology.

In our series, direct examination during surgical treatment confirmed pre-operative MRI findings in all patients; in two cases surgery better defined a solitary lesion of the biceps femoris muscle, previously indicated by MRI as a lesion of the common hamstring tendon. So, we consider MRI a mandatory investigation to get a correct diagnosis and satisfying preoperative planning; we do not recommend ultrasonography as the only preoperative investigation because of its low precision and sensitivity, and the need of a radiologist expert in this poorly understood but widely known injury. Due to the poor knowledge of PHT by many team physicians and sport health specialists, including orthopedic surgeons, often the time span between the onset of symptoms and the correct diagnosis is very long, and treatment is initially directed towards other pathologies [1, 7, 12].

In our experience, differential diagnostics must consider hamstring muscle tears, piriformis syndrome, ischiatic bursitis and chronic posterior compartment syndrome of the thigh; their localization, clinical manifestations and type of patients (young adult athletes) are the same for PTH. Then, sacroiliac joint pathologies and lumbosacral sciatica should be considered. More unusual pathologies are hematomas or soft-tissue tumours of the thigh (lipomas, sarcomas).

In hamstring muscle tears, pain is acute and localized more distally in the belly; often a painful palpable defect is present. Piriformis syndrome is characterized by buttock pain, rarely sciatica, leg weakness during exercise, resulting from sciatic nerve compression by the contracted and hypertrophied piriform muscle. Pain location, specific tests (Pace’s sign and Freiberg’s sign) and MRI findings demonstrating hypertrophy of the piriformis muscle are indicative.

In ischiatic bursitis, pain is located in gluteal region and evoked by palpation of the ischial tuberosity or by a prolonged sitting position. Unlike proximal hamstring tendinopathy, pain occurs at rest, disturbs sleep, and forces the patient to a continuous search for a more comfortable position.

Chronic posterior compartment syndrome of the thigh is another sport pathology caused by functional overload of hamstrings leading to their fast hypertrophy not followed by distension of their fascia; pain occurs during exercise, but not in the sitting position. In proximal hamstring tendinopathy nerve conduction studies are typically negative. Anyway, adhesions between the sciatic nerve and the thickened proximal hamstring tendons could cause episodic nervous compression; it typically occurs during stretching or the forward swing phase of running [1–3]. In our series 82 % of patients performed preoperative ENMG, resulting in return to normal in all cases. We consider ENMG useful uniquely to help in the differential diagnostics.

Proximal hamstring tendinopathy is an invalidating condition for patients, who are often high- level athletes that need a quick return to a complete performance status. Conservative treatment is the first therapeutic step, but its efficacy is controversial moreover because of lack of standardize treatment protocols [2]. According to the sole article that we could find in the literature, rehabilitation should be based on soft-tissue mobilization, frequent stretching, progressive eccentric works of hamstrings and core-stabilization exercises [7]. According to our experience and other studies, stretching of hamstrings only worsens symptoms [3]. Peritendinous corticosteroid injections should be effective in short-term pain relief, but often symptoms recur later, and are even more severe [3, 8]. Shockwave therapy is a promising technique, but it needs more investigations to understand in which cases, and when and how to use it [6].

When the diagnosis has been made, if the athlete already and unsuccessfully went through different treatment protocols, time wasting with additional conservative treatment is not advisable; operative treatment seems to be the sole solution to remove the cause of the disorder [3, 9].

Our surgical treatment lightly differs from those proposed by Young and Orava [9, 13]. We perform a partial transverse tenotomy of the affected tendon, which is thicker and involved in a fibrous sheath; then, a systematic sciatic nerve release is performed, from the ischial tuberosity to 10 cm more distally. Orava systematically cuts the lateral edge of the semimembranosus tendon 3–4 cm distal to its origin; then he sutures it to the biceps femoris tendon. He considers neurolysis not necessary. On the contrary, Young dissects the sciatic nerve along the lateral border of the proximal hamstrings till it is completely mobile; he removes any degenerative areas of the tendons with no attempt made to repair the defects.

We believe performing the tenotomy of the thickened tendon is enough to free the sciatic nerve and to release proximal hamstring at the origin. Moreover, analyzing the outcome of proximal hamstring injuries, Askling et al.[5] found that isolated proximal semimembranosus tendon injuries on average require a more prolonged recovery time than proximal biceps femoris injuries. Performing semimembranosus tenotomy is done only if it is the tendon afflicted, we believe we should try to avoid unnecessary prolonged recovery time in the cases in which the tendon involved is another one. Anyway, further studies are required to assess it.

This study has certain limitations. It is a retrospective case series, without a control group; most data were collected from medical records, but information about the postoperative period was obtained by a later questionnaire, which permitted collecting only subjective symptoms. A randomized controlled trial is needed to assess the optimal treatment.
